# DNA ligase IV syndrome; a review

**DOI:** 10.1186/s13023-016-0520-1

**Published:** 2016-10-07

**Authors:** Thomas Altmann, Andrew R. Gennery

**Affiliations:** 1 Institute of Cellular Medicine, Newcastle University, Newcastle upon Tyne, UK; 2 Great North Children’s Hospital, Newcastle upon Tyne, UK

**Keywords:** DNA Ligase 4, Severe combined immunodeficiency, Primordial dwarfism, Radiosensitive, Lymphoid malignancy

## Abstract

DNA ligase IV deficiency is a rare primary immunodeficiency, LIG4 syndrome, often associated with other systemic features. DNA ligase IV is part of the non-homologous end joining mechanism, required to repair DNA double stranded breaks. Ubiquitously expressed, it is required to prevent mutagenesis and apoptosis, which can result from DNA double strand breakage caused by intracellular events such as DNA replication and meiosis or extracellular events including damage by reactive oxygen species and ionising radiation.

Within developing lymphocytes, DNA ligase IV is required to repair programmed DNA double stranded breaks induced during lymphocyte receptor development.

Patients with hypomorphic mutations in *LIG4* present with a range of phenotypes, from normal to severe combined immunodeficiency. All, however, manifest sensitivity to ionising radiation. Commonly associated features include primordial growth failure with severe microcephaly and a spectrum of learning difficulties, marrow hypoplasia and a predisposition to lymphoid malignancy. Diagnostic investigations include immunophenotyping, and testing for radiosensitivity. Some patients present with microcephaly as a predominant feature, but seemingly normal immunity. Treatment is mainly supportive, although haematopoietic stem cell transplantation has been used in a few cases.

## Background

DNA ligase IV deficiency (OMIM 606593) or LIG4 syndrome (ORPHA99812), also known as Ligase 4 syndrome, is a rare autosomal recessive disorder characterised by microcephaly, abnormal facial features, sensitivity to ionizing radiation and combined immunodeficiency. Additional features can include developmental delay, bony deformations, skin conditions and susceptibility to malignancy (Table 1). It is caused by mutations in *LIG4* that encodes a key component of the ubiquitous non-homologous end-joining (NHEJ) pathway essential for the DNA double strand break (DSB) repair mechanism that is also utilized in the production of T and B lymphocyte receptors.

**Table 1:** List of reported presenting features in LIG4 patients [12–18, 22–27, 31]

Physical features	Microcephaly
	Growth restriction
	"Bird-like" or "Seckel syndrome-like" facies
	Bilateral epicanthic folds
	Hypogonadism
Bone abnormalities	Bone Hypoplasia
	Syndactyly
	Polydactyly
	Congential Hip Dysplasia
Skin conditions	Photosensitivity
	Psoriasis
	Eczema
	Widespread Ecchymosis
	Hypopigmentation
	Extensive Plantar Warts

## DNA damage and repair

DNA is constantly damaged in ways that, if left unrepaired, may lead to genetic sequence errors. Damaging factors include intracellular events such as DNA replication and meiosis, and extracellular events including damage by reactive oxygen species and ionising radiation. In order to maintain genomic integrity and stability, pathways have evolved to recognise and correct these errors. In mammalian cells the pathways include NHEJ, homologous recombination, base excision repair and DNA mismatch repair. Defects in any of the components of these pathways may allow DNA replication errors, such as addition, loss or rearrangement of genetic information. Errors of DNA-DSB repair are particularly damaging and may lead to mutagenesis causing carcinogenesis or premature cell death by apoptosis [1, 2]. Two DNA repair pathways have evolved to deal with these lesions. Homologous recombination utilises information from a homologous template to accurately repair breaks, when sister chromatids present readily available templates, generally limited to late S phase and G2 phase of the cell cycle in mammalian cells. When extensive homology is lacking NHEJ is the main DNA-repair pathway that mediates the joining of broken regions of DNA, and is the principle mechanism used in vertebrate cells during the G1 phase of the cell cycle. Individuals who harbour genetic mutations in components of DNA-DSB repair, demonstrate cellular sensitivity to ionising radiation and chemicals which induce DNA-DSB, and are more susceptible to developing oncogenic lesions.

As well as protecting genomic integrity, DNA-DSB repair pathways are utilised in adaptive immunity in the production of T- and B-lymphocytes. In order to counter any potential invading pathogen, a broad spectrum of T- and B-lymphocyte receptors must be produced to ensure they are able to recognise all possible threats [3]. This is ensured by variable, diverse and joining (V(D)J) recombination, a system of targeted DNA damage, in the form of programmed DNA-DSB and repair that has evolved to achieve this stochastically diverse T- and B-lymphocyte repertoire [2].

### DSB repair: NHEJ

The most rapid mechanism for the repair of DNA-DSB in mammalian cells is the NHEJ pathway. The classical NHEJ pathway, of which DNA Ligase IV is a critical component, is active in all stages of the cell cycle, but most active in G0 and G1 phases [4]. An alternative NHEJ pathway, most active in the S and G2 phases of the cell cycle, is dependent on signalling by poly(ADP-ribose) polymerase 1, utilizes microhomolgy, and is used when elements of the classical pathway are dysfunctional. Un-repaired DNA-DSB have a high probability of leading to mutagenesis and oncogenesis or apoptosis [4]. It is estimated that the average mammalian cell incurs 10 to 50 DNA-DSB per day, mostly by reactive oxygen species.

The presence of a DNA-DSB, generated through non-programmed events or programmed V(D)J recombination is recognised by a complex of which the constituent parts are MRE11, RAD50 and Nijmegen breakage syndrome protein 1 (Nibrin previously called NBS1) [4]. Ku70/80 subsequently binds the break and the DNA-Ku70/80 complex recruits the DNA dependant protein kinase catalytic subunit (DNA-PKcs) and activates the kinase activity. During this process, the DNA-Ku70/80-DNA-PKcs complex recruits proteins including Artemis, DNA ligase IV, XRCC4 and Cernunos-XLF to ligate the breakage site. Once the components are in place, DNA-PKcs is autophosphorylated and in turn phosphorylates Artemis. This enables Artemis/DNAPKcs to function as an endonuclease, leading to cleavage of 5’ and 3’ DNA overhangs. The DNA ligase IV/XRCC4/Cernunos-XLF complex AMP moiety temporarily attaches to the ends of the DNA and ensures ligation of the DNA-DSB [1, 2]. DNA-DSB often have complex ends so that the structure of aligned ends may prevent juxtaposition of strand break termini because ends have damaged or adducted nucleotides, mis-pairs, nucleotide gaps or hairpins. Polymerase μ fills gaps created by the breakage process to generate ends that can be efficiently ligated but adding complementary nucleotides after bridging noncomplementary ends [5, 6]. During V(D)J recombination terminal deoxynucleotidyl transferase (TdT), a polymerase structurally related to polμ adds nucleotides in a template-independent reaction, to increase junctional diversity at the V(D)J join, and thus increase the diversity of the lymphocyte receptor repertoire [7].

## Somatic recombination

The adaptive immune system is able to mount an effective immune response against a wide range of foreign pathogens, achieved by generation of an estimated 10^8^ cells each with their own unique antigen receptor that is able to recognise a single antigen-major histocompatibility complex (MHC) [8]. The process of stochastic rearrangement and re-joining of DNA sequences that code for the antigen recognition region of the receptors enables this diverse number of unique adaptive immune cells to be produced. This is known as V(D)J recombination and it is achieved by adapting pre-existing DNA damage repair mechanisms to repair the programmed DNA-DSB created during the recombination process. V(D)J recombination occurs in early T-lymphocyte thymic development, affecting α, β, γ and δ loci of the T Cell Receptor (TCR), and maturing B-lymphocytes in the bone marrow, affecting B Cell Receptor (BCR)/immunoglobulin heavy chain loci and immunoglobulin k and λ light chain loci.

### Events during V(D)J recombination

During G1 in the cell cycle, the RAG1/RAG2 complex induces site specific DNA-DSB at conserved non-coding DNA recombination signal sequence (RSS) on both sides of the site randomly targeted for recombination, forming two DNA ends:

1. Coding sequence ends formed as hairpin intermediates that reform immunoglobulin and TCR genes.
2. Blunt double stranded DNA non-coding signal ends that contain motifs for targeting site-specific DNA cleavage between the two RSS sites.

During this process ataxia-telangiectasia mutated (ATM) protein assists in stabilisation of the exposed DNA ends within the RAG complex. Nibrin, γH2Ax and 53BPI also migrate to the DNA-DSB and chromatin region of the recombining loci to stabilise the process [2]. Once the recombination has occurred the DNA-DSB are repaired by the NHEJ mechanism, described above.

If any of the NHEJ components are dysfunctional V(D)J recombination is not entirely lost. An alternative pathway exists utilising microhomology as a means to repair DSBs. However this pathway leads to a multitude of nucleotide deletions [9]. Theoretically the limited DNA repair that occurs in this alternative pathway could lead to production of a reduced number of unique antigen recognising receptors.

## LIG4 structure and models of LIG4

*Lig4* is located on chromosome 13q33–q34 [10]. Complete knockout of LIG4 in mice is embryological lethal and mutations described in humans are hypomorphic, leading to significantly impaired NHEJ but still maintaining some activity. Various murine models have confirmed the hypomorphic nature of LIG4 mutations seen in humans. One lacked a single copy of *LIG4*, and due to impaired NHEJ, cells showed excessive sensitivity to ionising radiation [11]. Human fibroblast cell lines developed from LIG4 patients also show significant radiosensitivity [12–18]. Nijnik and Rucci produced murine models of LIG4 syndrome that show great similarities with that of humans. Mice were immunodeficient; growth restricted and demonstrated progressive bone marrow failure as they aged [19, 20]. The cause of progressive bone marrow failure in LIG4 mouse models and humans is due to a progressive accumulation of DNA-DSB in haematopoietic pluripotent stem cells leading to cellular apoptosis [21].

## Epidemiology

Little is known of the prevalence of LIG4 syndrome. Globally only 28 cases have been described [12–18, 22–27] and additionally a small number of unpublished cases have been treated by haematopoietic stem cell transplant (HSCT) (personal communication, AR. Gennery). No formal estimate of prevalence has been made to date.

## Clinical presentation and complications

The first patient described with a *LIG4* mutation was developmentally and clinically normal with no microcephaly, but developed acute lymphoblastic leukaemia and following chemotherapy treatment developed profound leukopenia. Standard consolidation chemotherapy was omitted but prophylactic cranial radiotherapy proved devastating. He developed marrow hypoplasia, a desquamating rash over his scalp and bilateral mastoid radiation ulcers and died 8 months later from radiation-induced encephalopathy [25]. A fibroblast cell line, 180BR, was found to have defective DNA DSB repair. The authors investigated the NHEJ pathway in 180BR cells finding normal levels of DNA-PK, XRCC4 and DNA LIG4. Mutated DNA LIG4 in 180BR was unable to form stable enzyme-adenylate complexes. At high levels of ATP some complex forming activity was measured in 180BR, which may explain why the patient did not have any signs of overt immunodeficiency and had intact V(D)J recombination. However the defect was severe enough that 180BR was unable to repair radiation-induced DNA DSB for which maximum DNA Ligase IV activity may be required [15]. Since this first case was described, 27 further cases have been published with a broad spectrum of clinical features [12–18, 22–27].

**Fig. 1:**
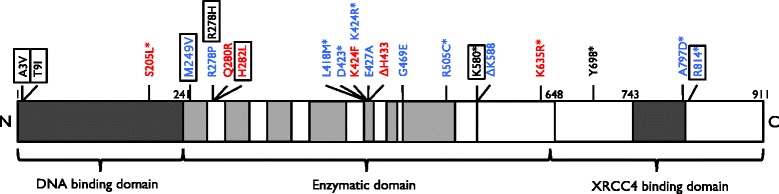
The structure of DNA LIG4 with the localisation of mutations found in patients with DNA LIG4 Syndrome. *Blue* colour denotes mutations that are associated with CID. *Red* denotes mutations that are associated with SCID. *Boxes* around mutations denote mutations that are associated with developing malignancies

### Physical features

The most common, although not universal, finding is that of congenital non-progressive microcephaly, described in 26 of the 28 patients. Sporadic physical developmental issues are described, the most common of which is severe growth restriction, which co-exists with microcephaly, and begins in-utero. Abnormal facial features are common with four patients described as “bird-like” or “Seckel syndrome-like” (beak-like nose, prominent mid-face, receding forehead and micrognathia) facial features and 13 with bilateral epicanthic folds and nose changes [24, 27]. Eight patients have been described with bone abnormalities including bone hypoplasia, syndactyly, polydactyly and congenital hip dysplasia. Three patients have been described with hypogonadism presenting with primary amenorrhea or failing to progress through puberty [23, 24, 27]. Ten patients presented with a variety of skin disorders including photosensitivity, psoriasis, eczema, erythroderma, widespread ecchymosis and hypopigmentation [16, 22–24, 27].

### Developmental features

A wide spectrum of neurodevelopmental delay is evident in LIG4 deficiency ranging from individuals who appear to have no developmental delay to those that suffer from profound learning difficulties [23, 28]. Microcephaly and neurodevelopmental delay are frequently present and characteristic. Microcephaly, which is evident prenatally, may result from emission of reactive oxygen species by rapidly replicating neurones in foetal development. These induce oxidative damage causing an accumulation of DNA-DSB, which causes irreversible collapse of DNA replication forks, halting neuronal cell development [29].

### Immunodeficiency

Due to disruption in V(D)J recombination, LIG4 syndrome is associated with immunodeficiency. Twelve patients have been reported with LIG4 syndrome after the finding of combined immunodeficiency (CID) with profound T- and B-lymphocytopenia and varying degrees of hypogammaglobulinaemia, often associated with a raised IgM due to defective isotype class switching, which also relies on programmed DNA-DSB. There is increased susceptibility to bacterial, viral and fungal infection leading to multiple hospital admissions and failure to thrive. The most severe form of immunodeficiency, severe combined immunodeficiency (SCID), has been described in four patients [12, 14, 18, 23]. Additionally, one patient has been described with SCID and features of Omenn’s syndrome [22], more frequently associated with hypomorphic RAG1/2 defects leading to abrogated V(D)J recombination [30]. Autoimmunity was described in one patient [9].

### Malignancy

Defective DNA-DSB repair represents a substantial risk factor for mutagenesis and the development of malignancy. Six LIG4 patients have been reported in the literature, presenting with malignancy. Lymphoma affected three patients including two with Epstein-Barr virus associated lymphoma [13, 26, 31, 32]. One patient with Dubowitz syndrome developed malignant squamous cell carcinoma and was diagnosed with a *LIG4* mutation retrospectively [16].

## Phenotype-genotype correlations

LIG4 syndrome has a broad clinical presentation and phenotype. Most cases described to date have missense *LIG4* mutations or nucleotide deletions displaying a range of phenotypic presentations [12–15, 24, 26, 27]. The missense mutations lead to 5-10 % of LIG4 function: these patients present with CID [12, 13, 22, 26]. Truncating (nonsense) mutations, depending on location within the gene, present with a range of signs (Fig. 1). Some present with no evidence of immunodeficiency and others with SCID. Murray et al. [23] found that a genotype–phenotype correlation was seen with the position of truncating mutations corresponding to disease severity. The authors found that the “early” truncating mutations, which caused the addition of an early stop codon that produced the shortest of the proteins, resulted in the most severe phenotypes with these patients developing SCID [23].

## Diagnosis

Diagnosis of LIG4 is initiated by clinical suspicion. Clinical findings of the main characteristics, namely microcephaly, combined immunodeficiency with or without developmental delay, are sufficient to confirm a rare immunodeficiency possibly involving defective DNA repair pathways. Clinical laboratory features that increase suspicion include marrow hypoplasia with anaemia and thrombocytopenia, lymphocytopenia with marked B-lymphocytopenia, panhypogammaglobulinaemia or evidence of isotype class-switching impairment with raised IgM and absent or low IgA and IgG.

In T- and B-lymphocytes from patients, there is biochemical evidence of reduced class switch recombination with increased use of microhomology-mediated end-joining at switch junctions and in *TRB* and *IGH* junctions and use of long microhomology. Additionally, there is a lack of Sμ-α junctional mutations [32, 33]]. In the T- and B-lymphocyte receptor, there may be restriction of CDR3 length and diversity [30]. In experimental cell models, a small reduction in V(D)J frequency has been found, with a significant reduction in fidelity of signal joins [21].

In many patients, there is increased chromosome 7:14 translocation on karyotype analysis. Clonogenic survival assays confirm radiosensitivity, performed by subjecting fibroblasts from the patient to increasing doses of ionising radiation and measuring percentage survival of the cells after a fixed time period [34]. Delayed DNA-dsb repair kinetics can be measured by assessing appearance and resolution of γH2AX foci in irradiated cells [35]. The frequency of V(D)J recombination [36] and fidelity of signal joint formation [37] can be assessed using recombination substrates in a fibroblast cell line generated from the patient.

Once radiosensitivity is confirmed, specific genetic testing for *LIG4* and other DNA repair genes can be performed. To date, no patients with mutations in *LIG4* have been described who do not exhibit sensitivity to ionising radiation.

### Modified clinical presentations

Until recently LIG4 syndrome was only identified through genetic testing after the diagnosis of immunodeficiency and/or malignancy in microcephalic patients. Murray et al. observed this and took the reverse approach to diagnosis. The authors screened 138 patients with microcephalic primordial dwarfism for DNA Ligase IV mutations. Eleven microcephalic primordial dwarfism patients were identified as having LIG4 syndrome all off whom had cellular radiosensitivity. Nine developed cytopenia due to bone marrow failure requiring transfusion, seven of whom developed cytopenia post-LIG4 screening. Interestingly no patients were diagnosed with CID before screening and only one patient was diagnosed with SCID. Retrospectively a further six patients showed signs of CID, most with increased infection rate, low B-lymphocyte numbers and hypogammaglobulinaemia. Four required HSCT due to significant immunodeficiency. This may be due to the accumulation of DNA-DSB, and therefore progressive apoptosis, in haematopoietic stem cells of LIG4 patients causing progressive immunodeficiency as lymphocytogenesis reduces over time [21]. None of the patients identified through the authors screening had developed malignancy [23], possibly indicating that malignancy in LIG4 syndrome is a late feature of the disease. As with an increasing number of immunodeficiencies, mild phenotypes with a lack of genotype/phenotype correlation may be encountered. A family with LIG4 syndrome has recently been described in which three mutated siblings showed cellular and molecular features of the disease, but two were asymptomatic, indicating that within families the phenotype can vary dramatically [38].

## Differential diagnosis

A number of conditions have features that overlap with LIG4 syndrome. Nijmegen breakage syndrome (NBS) patients harbour *NBN* mutations, the product of which, nibrin, is involved in recognising DNA-DSB [4]. This leads to similar “bird-like” facial features, microcephaly and neurodevelopmental delay as exhibited by LIG4 patients. NBS patients are prone to respiratory infections due to T-lymphocytopenia and variable hypogammaglobulinaemia. Autoimmunity is described in a few patients. Almost 50 % show a predisposition to lymphoid malignancy [39].

A few patients with Cernunnos-XLF deficiency are described with mutations in *NHEJ1*, who present with similar physical and neurodevelopmental features, CID with T- and B-lymphocytopenia, isotype class switching defects and recurrent infections. Cernunnos-XLF patients, like LIG4, have also co-presented with bony malformations [2]. Cernunnos-XLF is a core component of the NHEJ complex and interacts closely with LIG4 during the DNA-DSB ligation process.

The third enzyme that makes up the NHEJ ligation enzyme complex is XRCC4, which interacts strongly with LIG4 through a tandem BRCA1 carboxyl terminal domain in LIG4 and a coiled-coil region in XRCC4, to form a highly stable complex. A number of patients have recently been reported with mutations in XRCC4. Phenotypically, the clinical presentation is similar to patients with LIG4 syndrome, with characteristic microcephaly and neurodevelopmental delay. Interestingly, given that XRCC4 is required to stabilise LIG4, it is surprising that to date, none of the patients described exhibit clinical immunodeficiency, despite the marked DNA-DSB repair defect. A molecular alteration in repair pattern is described during class switch recombination, but V(D)J recombination appears normal [40].

Fanconi anaemia is characterized by bone marrow failure, often accompanied by other anomalies including skeletal, renal, cardiac and gastrointestinal defects, skin hypo-pigmentation and predisposition to malignancy, particularly leukemia. Most immunological manifestations relate to bone marrow failure, but some patients present in infancy or early childhood with significant or prolonged infections, more consistent with immunodeficiency [41]. Microcephaly is not a feature of Fanconi anaemia. Laboratory assessments include a diepoxybutane or mitomycin C chromosome fragility test of blood lymphocytes. Fifteen genes associated with Fanconi anaemia have been identified, that have a role in repairing DNA inter-strand crosslink damage. Cells generally show hypersensitivity to agents that cause DNA inter-strand crosslinks, but a few also demonstrate sensitivity to ionising radiation [42]. Fanconi anaemia proteins do not have direct a role in lymphocyte receptor development or modification. The immunological effects more likely result from the effects of inter-strand DNA crosslinks occurring during cellular development, which lead to bone marrow failure.

ATR-Seckle Syndrome which also presents with microcephaly and “bird-like” facial features occurs due to abnormal function in ATR, a protein that monitors single stranded DNA replication errors at replication forks [43]. However cases reported have a normal immunological profile although some have developed lymphoid malignancies [28, 44]. The crucial biochemical difference between ATR-Seckle and LIG4 is that ATR-Seckle cells do not display radiosensitivity to ionising radiation.

## Treatment

Initial treatment of LIG4 syndrome is supportive, with haematological support of marrow hypoplasia as required, longterm antibiotic, antiviral and antifungal chemoprophylaxis and immunoglobulin substitution. Patients remain at risk of severe infections because of chemo-prophylaxis resistance or compliance issues, and the risk of lymphoid malignancy increases with time. Excessive exposure to ionising radiation should be avoided where possible, and radiographic and computerised tomography should be avoided where possible. Haematopoietic stem cell transplantation is a curative treatment for CID and SCID immunophenotypes, and might reduce the long-term risk of developing lymphoid malignancy due to improved tumour surveillance. Because of the radiosensitivity displayed by LIG4, conditioning regimes should not include irradiation. A low intensity or modified Fanconi anaemia-based conditioning regimen may give the best possible survival, and restoration of normal immunity and marrow hypoplasia, and alkylating agents should be avoided [45]. Longterm effects of this approach need to be determined, and there may be a risk of secondary tumours given the systemic nature of LIG4 deficiency. Determining optimum treatment for LIG4 deficiency should be assessed on an individual basis. Factors such as immunological profile, infection rate and severity, missed school days and dependency on blood products, amongst others, should be considered when choosing the most appropriate treatment. It should be noted that HSCT has no effect on microcephaly or neurodevelopmental delay in these patients [22].

Information on haematopoietic stem cell transplantation is available on ten patients [12–14, 17, 22, 24, 27, 46] and has been successful in four cases. Four patients died (2 from multi-organ failure during the conditioning period, one from Epstein Barr virus-driven post-transplantation lymphoproliferative disease, and one from hepatic veno-occlusive disease), all of whom received alkylating agents; six patients have survived, three received reduced intensity conditioning.

Excellent social care ensures LIG4 syndrome patients maintain a good quality of life. Extra support is required for parents with children with neurodevelopmental delay, children benefit from attending schools with a positive environment for people with learning difficulties [17].

## Conclusions

LIG4 syndrome is an extremely rare condition characterised by microcephaly, abnormal “bird-like” facial features, neurodevelopmental delay and immunodeficiencies with radiosensitivity. Specific features that should raise diagnostic suspicion include prenatal microcephaly with growth retardation and developmental delay, marrow hypoplasia, recurrent infection with lymphocytopenia, and hypogammaglobulinaemia, often with a raised IgM. Haematopoietic stem cell transplantation for immunodeficiency can be curative but it is not without its complications, and reduced intensity conditioning regimens should be utilised, with omission of radiotherapy. In both human cases and mouse models, bone marrow failure and immunodeficiencies can be progressive. It may be beneficial to screen patients with clinical characteristics of LIG4 syndrome in order to diagnose and potentially treat immunodeficiency before development of significant sequalae in order that early intervention with antimicrobial prophylaxis, immunoglobulin replacement and possibly HSCT can be considered.

## Abbreviations

ATM: ataxia telangiectasia mutated
ATP: Adenosine triphosphate
ATR: ataxia-telangiectasia and RAD3-related
BCR: B cell receptor
BRCA1: Breast Cancer gene 1
CDR3: complimentary determining region 3
CID: combined immunodeficiency
DNA-PK(cs): DNA protein kinase (catalytic subunit)
DSB: double strand break
γH2AX: γH2A histone family, member X
HSCT: haematopoietic stem cell transplantation
*IGH*: immunoglobulin heavy locus
MHC: major histocompatibility complex
MRE11: Meiotic recombination 11 protein
NHEJ: non-homologous end joining
NBS: Nijmegen breakage syndrome
RAD50: radiation-sensitive DNA repair protein 50
RAG: recombination activating gene
RSS: recombination signal sequence
SCID: severe combined immunodeficiency
TCR: T cell receptor
TdT: Terminal deoxynucleotidyl transferase
*TRB*: T cell receptor beta locus
V(D)J: variable, diverse, joining
XRCC4: X-ray repair, complementing defective, in Chinese hamster, 4
XLF: XRCC4-like factor
